# The prevalence and incidence, resource use and financial costs of treating people with attention deficit/hyperactivity disorder (ADHD) in the United Kingdom (1998 to 2010)

**DOI:** 10.1186/1753-2000-7-34

**Published:** 2013-10-11

**Authors:** Sarah E Holden, Sara Jenkins-Jones, Chris D Poole, Christopher Ll Morgan, David Coghill, Craig J Currie

**Affiliations:** 1 Primary Care and Public Health, School of Medicine, The Pharma Research Centre, Cardiff Medicentre, Cardiff University, Cardiff CF14 4UJ, UK; 2 Global Epidemiology, Pharmatelligence, Cardiff Medicentre, Cardiff CF14 4UJ, UK; 3 Division of Neuroscience, Medical Research Institute, University of Dundee, Dundee DD1 9SY, UK

**Keywords:** ADHD, CPRD, Prevalence, Incidence, Healthcare cost

## Abstract

**Background:**

Attention deficit/hyperactivity disorder (ADHD) is a common disorder that often presents in childhood and is associated with increased healthcare resource use. The aims of this study were to characterise the epidemiology of diagnosed ADHD in the UK and determine the resource use and financial costs of care.

**Methods:**

For this retrospective, observational cohort study, patients newly diagnosed with ADHD between 1998 and 2010 were identified from the UK Clinical Practice Research Datalink (CPRD) and matched to a randomly drawn control group without a diagnosis of ADHD. The prevalence and incidence of diagnosed ADHD were calculated. Resource utilisation and corresponding financial costs post-diagnosis were estimated for general practice contacts, investigations, prescriptions, outpatient appointments, and inpatient admissions.

**Results:**

Incidence of diagnosed ADHD (and percentage change using 1998 as a reference) increased from 6.9 per 100,000 population in 1998 to 12.2 per 100,000 (78%) in 2007 and then fell to 9.9 per 100,000 (44%) by 2009. The corresponding prevalence figures were 30.5, 88.9 (192%) and 81.5 (167%) per 100,000. Incidence and prevalence were higher in males than females. Mean annual total healthcare costs were higher for ADHD cases than controls (£1,327 versus £328 for year 1, £1,196 vs. £337 for year 2, £1,148 vs. £316 for year 3, £1,126 vs. £325 for year 4, and £1,112 vs. £361 for year 5).

**Conclusions:**

The prevalence of diagnosed ADHD in routine practice in the UK was notably lower than in previous reports, and both prevalence and incidence of diagnosed ADHD in primary care have fallen since 2007. Financial costs were more than four times higher in those with ADHD than in those without ADHD.

## Background

Attention deficit/hyperactivity disorder (ADHD) is common and more likely to affect boys than girls, with an estimated prevalence in the UK of 3.6% and 0.9%, respectively, in children aged 5–15 years, using DSM-IV criteria [1]. Anecdotally, there is a commonly held belief that the prevalence of ADHD has risen markedly over the previous 20 years, with a corresponding increase in the financial cost of medicines indicated for ADHD [2,3]. ADHD is a chronic condition that is often associated with significant impairments in academic performance and social functioning [4,5]. Over 65% of those with ADHD also have one or more comorbid disorders. These include dyslexia, developmental coordination disorder, Tourette’s syndrome, autistic spectrum disorders, conduct and oppositional defiant disorders, and substance abuse [4,6]. ADHD is also associated with disrupted parent–child relationships and increased parent stress levels [4,7]. Treatment costs for patients with ADHD are greater than those without [8-15].

In the UK, the National Institute for Health and Care Excellence (NICE) has recommended that diagnosis of ADHD and treatment initiation should be conducted within secondary care [16]. When medication is used the dose should also be titrated and stabilised by a specialist. Once the patient is stabilised on treatment, prescribing and monitoring can be carried out in primary care under a shared-care protocol [16]. Whilst the popular press frequently comments on increased rates of diagnosis of ADHD and questions whether ADHD is over-diagnosed and over-treated [17], data from reviews of clinical practice suggest the opposite may be true with ADHD being both under recognised and under treated [18]. There are, however, few studies characterising the epidemiology of diagnosed ADHD in the UK and the healthcare cost to the NHS of treating children both with and without ADHD.

The aim of this retrospective, observational cohort study was to characterise the incidence and prevalence of diagnosed ADHD and to determine the corresponding resource use and financial cost of care for children, adolescents, and adults with ADHD compared with a matched control group over a 12-year period to 2010.

## Methods

### Data sources

Data were extracted from the CPRD (Clinical Practice Research Datalink) [19]. CPRD contains clinically rich data collected in a non-interventional manner from the daily record-keeping of primary-care physicians in the UK. These data include demographics, medical history, test results, outpatient letters, and prescriptions. There are, in total, 143 million acceptable person-years of computerised data in CPRD, and the dataset is broadly representative of the UK population. Following record-linkage to NHS hospital episode statistics (HES), CPRD additionally contains details of inpatient admissions for a proportion of practices in England. The data extract used in this study includes records up to June 2012. Ethical approval for this study was granted by the CPRD Independent Scientific Advisory Committee on 1st March 2012, protocol number 12_025R2.

### Study population

#### Cases

Patients were selected from CPRD if they had received two or more diagnoses for ADHD in their clinical history, or they had received at least one diagnosis of ADHD and at least one prescription for a medicine licensed for the management of ADHD. For cases where there was no prescription for an ADHD medication, the requirement of two or more diagnoses was used to avoid selecting for patients with only a provisional diagnosis recorded by the GP prior to assessment by a specialist. Under NICE guidelines, diagnosis should be made by a mental health specialist; therefore the second diagnosis is used to confirm that the patient has ADHD. A medicine used for the management of ADHD was defined as a product containing one of the following drugs: dexamfetamine, methylphenidate or atomoxetine. Pemoline (indicated for hyperkinetic syndrome but not generally available in the UK after 1997) [20] and modafinil (not licensed for the management of ADHD nor for use in children) [21] were not used for case selection. The study index date was the date of ADHD presentation, taken as the earlier of their first recorded diagnosis date for ADHD or their first prescription for a medicine used in the management of ADHD.

Cases were excluded from the analysis if they had a history of narcolepsy. In order to identify incident cases only, cases with less than six months’ “wash-in” for relevant parameters were also excluded (Additional file 1: Figure S1). No exclusion criteria based on age were implemented; however, the results were split by age group (0–5, 6–17 and ≥18 years) because licensed and recommended treatments vary by age. For example, atomoxetine and methylphenidate are not licensed in children younger than 6 years. In addition, the NICE guidelines do not recommend pharmacological treatment in preschool children. After school leaving age (≤18), NICE recommends that patients should be reassessed before transfer to adult services to ensure that continuing treatment into adulthood is still warranted and to facilitate transition. In addition, only atomoxetine is licensed for the treatment of ADHD in adults.

#### Controls

The healthcare costs and resource use of the ADHD group were compared to a randomly drawn control group of patients matched on year of birth, gender and GP practice. Control patients had no history of ADHD and had received no prescription for a medication indicated for ADHD.

Cases and controls were included in an annual cost calculation if they had a complete year’s observation for the year in question in both CPRD and CPRD-linked HES. Therefore, patients were excluded from the analysis of the costs for year 1 if they had less than 12 months’ observations from the index date to the last date of any prescription or the censor date, whichever was earlier. For year 2, patients were excluded if they did not have a complete year of data from 366 days to 730 days following their index date. The same rule was applied for the calculation of costs for years three through five.

### Diagnostic incidence of ADHD

The incidence of diagnosed ADHD was calculated by dividing the number of new cases of ADHD each year by the number of person-years at risk in the CPRD data set for the same year (including those registered but with no GP attendance).

#### Denominator

The number of person-years of people without ADHD was calculated by adding the number of days each patient had been present in the CPRD database for each specific year. Patients were included in the denominator until the earliest of their death date, transferred-out date, or ADHD presentation date. Patients who did not meet the selection criteria for the study were included in the denominator data.

#### Numerator

On the date of ADHD presentation, cases were included in the numerator portion of the incidence calculation for that specific year.

The incidence of treated ADHD was calculated using the same method. For calculations of incidence by gender and age group only those patients of the appropriate age or gender were included in the numerator and denominator parts of the incidence calculation.

### Diagnostic prevalence of ADHD

The point prevalence of diagnosed ADHD was calculated each year by dividing the number of patients with ADHD on 1st July (mid-year point) of that year by the total number of patients registered in CPRD on that date.

#### Numerator

A patient was included as a prevalent case if they met the selection criteria for the study, their ADHD presentation date was prior to 1st July of the specific year, and the later of their last ADHD diagnosis or last prescription for an ADHD medication was after 1st of July of that year. However, in order to allow for an adequate washout period (more than 12 months), prevalence was only calculated from 1998 to 2009. A washout period was considered necessary as the chance of receiving a diagnosis for ADHD following the mid-year point reduces as the time between the mid-year point and the last collection date for the database becomes shorter.

#### Denominator

This was the total number of patients registered in CPRD on 1st July of the specific year.

For calculations of prevalence by gender and age group only those patients of the appropriate age and gender were included in the numerator and denominator parts of the prevalence calculation.

### Estimation of the cost of healthcare in CPRD

Resource use and costs were applied to the following areas of patient care: prescriptions, primary-care contacts, investigations, hospital admissions, and outpatient appointments. The aim was to calculate the overall cost of treating an individual with ADHD not just the cost of treating the ADHD itself. Annual costs for the first five years following the index date were estimated.

### Prescription costs

Each prescription item listed in CPRD was attributed a net ingredient cost (NIC) from the corresponding year of the Prescription Cost Analysis (PCA) [22,23]. The NIC refers to the cost of the drug before discounts and does not include any dispensing costs or fees [24]. All NICs were adjusted for inflation to 2011 prices [25]. Either an exact match was made or the British National Formulary (BNF) taxonomy was utilised to attribute an average NIC per item for the BNF sub-paragraph, section or chapter.

### Outpatient attendance costs

Outpatient events were identified from CPRD’s consultation table if they had a consultation type indicative or suggestive of an outpatient appointment. The outpatient department and whether the consultation was a first or follow-up visit were used to allocate each appointment to an outpatient tariff [26].

### Cost of investigations

Investigations were identified, including both pathology and diagnostic services. Several reference sources were used to attribute a cost to these tests [26-29]. Laboratory tests carried out on the same day were grouped into test panels where appropriate in order to take account of any reduction in cost of carrying out more than one test at the same time [30].

### Primary-care consultations

Each consultation was classified by consultation type (e.g. surgery appointment, clinic, home visit, telephone consultation) and staff type (e.g. GP, practice nurse, mental health nurse, district nurse) and then assigned an average cost as listed in the Unit Cost of Health and Social Care 2010 from the Personal Social Services Research Unit (PSSRU) [31]. Where average cost per hour was the only cost published in the Unit Costs of Health and Social Care, the UK GP workload survey [32] was utilised in order to determine the average length of the consultation. From this figure, the average cost per consultation could be calculated.

### Hospital admissions

CPRD-linked HES records allowed us to cost inpatient admissions. From the care pathway outlined in the NICE guidelines for ADHD, we would not expect patients to be routinely admitted to hospital as a direct result of their ADHD [16]. However, children and adolescents with ADHD may be more prone to other problems requiring admission such as accidents or self-harm [16].

Data from inpatient admissions recorded in HES were processed into Healthcare Resource Groups (HRGs) using HRG-4 grouper [33]. The HRGs were then matched to NHS Reference Costs 2009–2010 [34]. It was not possible to differentiate between elective or emergency day-case admissions from the data available, and so costs were averaged by ratio of each admission type. Data on procedures were not available and so all costs were inflated by 17.5%: the average difference between procedural and non-procedural admissions.

## Results

3,229 cases with ADHD and 7,429 matched control patients were identified in CPRD (Table 1). The mean age at diagnosis was 10.4 (sd 5.9) years for cases and 10.4 (6.1) years for controls, and 85% of cases and 86% of controls were male. Baseline characteristics are detailed in Table 1.

**Table 1:** Baseline characteristics for cases and controls

**Age group**	**Parameter**	**Cases**	**References**
All ages	N	3,229	7,429
	Males, n (%)	2,759 (85%)	6,354 (86%)
	Females, n (%)	470 (15%)	1,075 (14%)
	Age, mean (sd), years	10.4 (5.9)	10.4 (6.1)
Aged 6 to 17 years at index date	N	2,873	6,598
	Males, n (%)	2,487 (87%)	5,707 (86%)
	Females, n (%)	386 (13%)	891 (14%)
	Age, mean (sd), years	9.8 (2.8)	9.8 (2.8)
Aged ≥18 years at index date	N	141	300
	Males, n (%)	86 (61%)	183 (61%)
	Females, n (%)	55 (39%)	117 (39%)
	Age, mean (sd), years	31.7 (10.7)	33.2 (12.3)

### Incidence and prevalence of diagnosed ADHD

**Figure 1:**
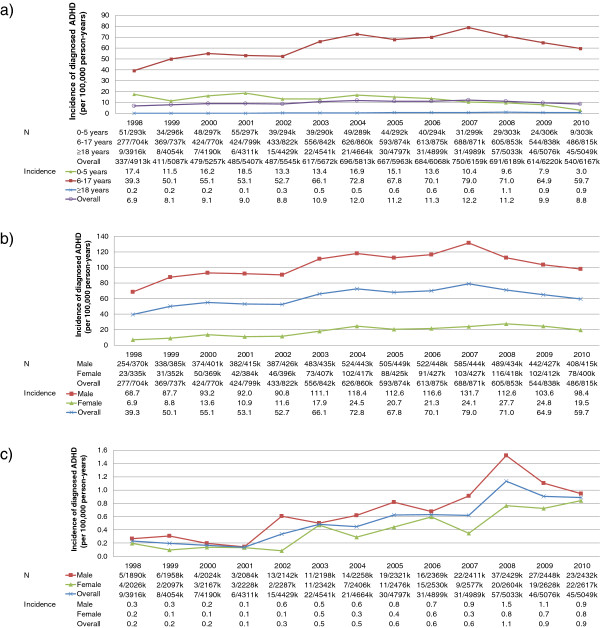
Incidence of ADHD (per 100,000 person-years) in the UK between 1998 and 2010 a) by age group, b) for patients aged 6–17 years by gender and c) Patients aged ≥18 years by gender.

**Figure 2:**
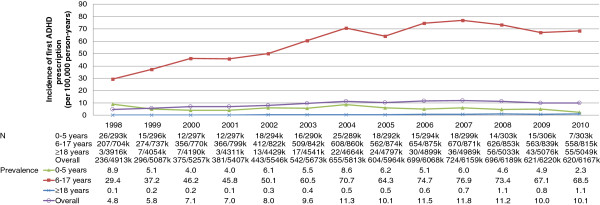
Incidence of first prescription for ADHD medication (per 100,00 person-years) by age group between 1998 and 2010.

In 1998, the annual incidence of diagnosed ADHD across all ages was 6.9 cases per 100,000 population (per100k; Figure 1a). This peaked in 2007, with 12.2 cases per100k (an increase of 78%). Overall, the incidence of diagnosed ADHD had fallen by 2010 to 8.8 per100k (an increase of 28% relative to 1998). The incidence of diagnosed ADHD in children and adolescents aged 6 to 17 years increased from 39.3 per100k in 1998 to 79.0 per100k (101% increase using 1998 as a reference) in 2007 before decreasing to 59.7 per100k (52% increase from 1998) in 2010 (Figure 1b). In 1998, the incidence of ADHD was 10 times higher in males than in females for patients aged 6 to 17 years but only five times higher in 2010. The incidence rate in adults was much lower than for patients aged 6 to 17 years and increased from 0.2 per100k in 1998 to 1.1 per100k (393% increase from 1998) in 2008 before falling to 0.9 per100k (288%) in 2010 (Figure 1c). For adults, the incidence rate in males was 1.3 times higher than in females in 1998 but only 1.1 times higher in 2009. The incidence of treated ADHD was 4.8 per100k in 1998 and reached a peak of 11.8 per100k (145%) in 2007 (Figure 2) before decreasing to 10.1 per100k (109%) in 2010.

**Figure 3:**
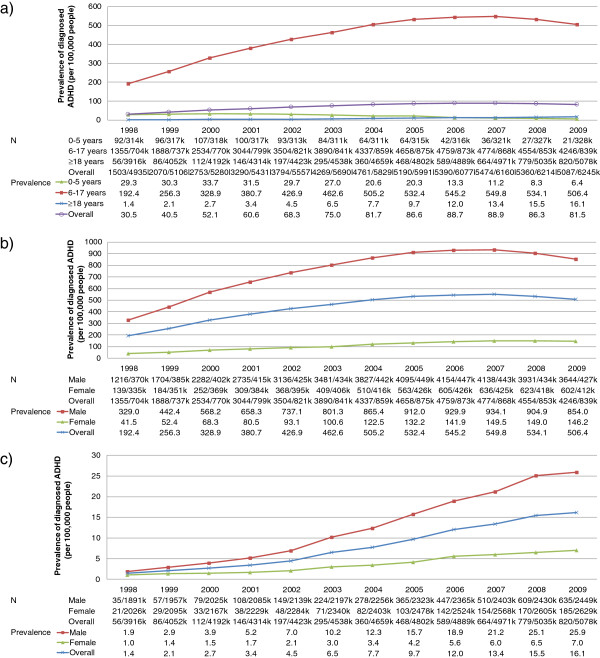
Prevalence of ADHD (per 100,000) in the UK between 1998 and 2009 a) by age group, b) for patients aged 6–17 years at index date by gender and c) for patients aged ≥18 years at index date.

The overall prevalence (and percentage change using 1998 as the reference) of diagnosed ADHD increased from 30.5 per100k in 1998 to 88.9 per100k (192%) in 2007 (Figure 3a). The prevalence then fell to 81.5 per100k (167%) in 2009 (Figure 3a). The diagnosed prevalence of ADHD was much higher in children aged 6 to 17 years than in adults. However, the prevalence increased in both groups between 1998 and 2007. In 1998, the diagnosed prevalence of ADHD was 192.4 per100k patients aged 6 to 17 years and 1.4 per100k in adults. By 2007, the prevalence was 549.8 per100k (186% change from 1998) in patients aged 6 to 17 years and 13.4 per100k (834%) in adults. By 2009, the prevalence of diagnosed ADHD in patients aged 6 to 17 years had fallen to 506.4 per100k (163%) but continued to increase to 16.1 per100k (1,029%) in adults (Figure 3a). The prevalence of diagnosed ADHD was 7.9 and 1.8 times higher in males than in females for patients aged 6 to 17 (Figure 3b) and adults (Figure 3c), respectively, in 1998 and 5.8 and 3.7 times higher in 2010.

### Resource use and costs

**Figure 4:**
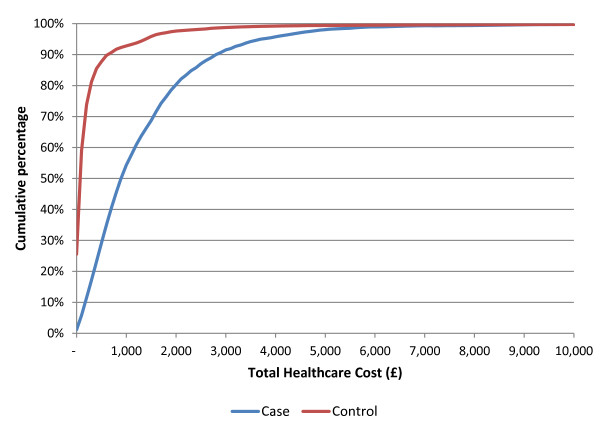
Distribution of healthcare costs in the first year following index date.

**Figure 5:**
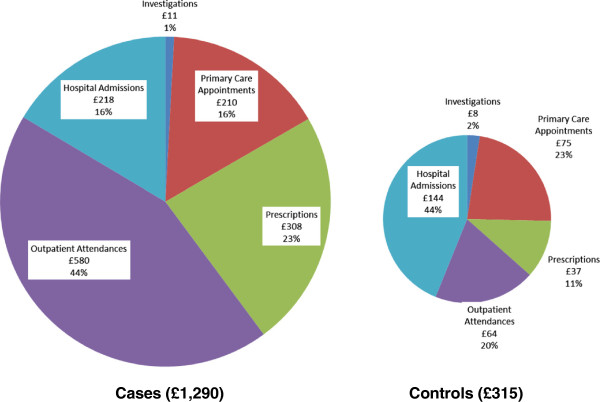
Breakdown of average annual costs (all ages) in the first year following index date.

All healthcare costs were positively skewed, particularly in the control group (Figure 4). Total annual cost ranged from £0 per year to £132,765 for the control group and £0 to £91,891 for cases. 26% of controls and 1% of cases incurred no healthcare costs at all in the first year, where the mean cost was four times higher for cases (£1,327 [sd £2,114] vs. £328 [sd £2,248], p < 0.001; Table 2). The median cost (inter-quartile range) was lower than the mean cost in both groups at £890 (£427–£1,742) vs. £69 (£0–£214) for cases and controls, respectively. Outpatient attendances accounted for 44% of costs for cases vs. 20% for controls (Figure 5). Specific costs were as follows: investigations (£11 vs. £8), primary-care appointments (£210 vs. £75), prescriptions (£308 vs. £37), outpatient attendances (£580 vs. £64), and hospital admissions (£218 vs. £144). Resource use is listed in Table 3.

**Table 2:** Total NHS healthcare costs for cases and controls in the first year following index date

**Age group**	**Resource Type**	**Group**	**Mean**	**Standard Deviation**	**Median**	**Percentile 25**	**Percentile 75**
All ages	Investigations	Case	£11	£38	£0	£0	£0
		Control	£8	£35	£0	£0	£0
	Primary-Care Appointments	Case	£210	£187	£166	£81	£279
		Control	£75	£100	£31	£0	£93
	Prescriptions	Case	£308	£384	£185	£53	£422
		Control	£37	£312	£2	£0	£15
	Outpatient Attendances	Case	£580	£882	£0	£0	£906
		Control	£64	£255	£0	£0	£0
	Hospital Admissions	Case	£218	£1,770	£0	£0	£0
		Control	£144	£2,068	£0	£0	£0
	Total	Case	£1,327	£2,114	£890	£427	£1,742
		Control	£328	£2,248	£69	£0	£214
Aged 6 to 17 years at index date	Investigations	Case	£10	£34	£0	£0	£0
		Control	£8	£34	£0	£0	£0
	Primary-Care Appointments	Case	£199	£171	£155	£73	£279
		Control	£70	£92	£31	£0	£93
	Prescriptions	Case	£306	£363	£192	£57	£423
		Control	£37	£326	£2	£0	£14
	Outpatient Attendances	Case	£572	£865	£0	£0	£899
		Control	£62	£253	£0	£0	£0
	Hospital Admissions	Case	£203	£1,838	£0	£0	£0
		Control	£139	£2,171	£0	£0	£0
	Total	Case	£1,290	£2,119	£879	£425	£1,689
		Control	£315	£2,354	£64	£0	£198
Aged ≥18 years at index date	Investigations	Case	£42	£93	£2	£0	£38
		Control	£24	£67	£0	£0	£17
	Primary-Care Appointments	Case	£375	£326	£298	£186	£478
		Control	£137	£188	£75	£31	£186
	Prescriptions	Case	£488	£580	£304	£113	£603
		Control	£65	£242	£6	£0	£36
	Outpatient Attendances	Case	£614	£1,065	£87	£0	£753
		Control	£83	£316	£0	£0	£0
	Hospital Admissions	Case	£324	£1,002	£0	£0	£0
		Control	£241	£1,065	£0	£0	£0
	Total	Case	£1,844	£2,118	£1,185	£648	£2,365
		Control	£550	£1,460	£130	£31	£427

**Table 3:** NHS Healthcare resource use for cases and controls in the first year following index date

**Age group**	**Resource type**	**Group**	**Mean**	**Standard deviation**	**Median**	**Percentile 25**	**Percentile 75**
All ages	Investigations	Case	1.3	4.4	0.0	0.0	0.0
		Control	0.8	3.9	0.0	0.0	0.0
	Primary-Care Appointments	Case	6.8	5.9	5.0	3.0	9.0
		Control	2.4	3.1	1.0	0.0	3.0
	Prescriptions	Case	11.0	11.7	9.0	4.0	14.0
		Control	2.9	7.1	1.0	0.0	3.0
	Outpatient Attendances	Case	2.6	4.1	0.0	0.0	4.0
		Control	0.4	1.3	0.0	0.0	0.0
	Hospital Admissions	Case	0.1	1.1	0.0	0.0	0.0
		Control	0.1	0.7	0.0	0.0	0.0
Aged 6 to 17 years at index date	Investigations	Case	1.1	4.1	0.0	0.0	0.0
		Control	0.7	3.7	0.0	0.0	0.0
	Primary-Care Appointments	Case	6.4	5.4	5.0	2.0	9.0
		Control	2.2	2.8	1.0	0.0	3.0
	Prescriptions	Case	10.5	9.6	9.0	4.0	14.0
		Control	2.7	6.8	1.0	0.0	3.0
	Outpatient Attendances	Case	2.5	4.0	0.0	0.0	4.0
		Control	0.3	1.3	0.0	0.0	0.0
	Hospital Admissions	Case	0.1	1.2	0.0	0.0	0.0
		Control	0.1	0.7	0.0	0.0	0.0
Aged ≥18 years at index date	Investigations	Case	5.4	8.9	1.0	0.0	9.0
		Control	3.6	8.2	0.0	0.0	2.0
	Primary-Care Appointments	Case	12.4	10.5	10.0	6.0	16.0
		Control	4.3	5.8	2.0	1.0	6.0
	Prescriptions	Case	21.1	28.1	11.0	5.0	24.0
		Control	5.1	12.2	1.0	0.0	4.0
	Outpatient Attendances	Case	2.8	4.8	1.0	0.0	4.0
		Control	0.4	1.5	0.0	0.0	0.0
	Hospital Admissions	Case	0.2	0.7	0.0	0.0	0.0
		Control	0.1	0.5	0.0	0.0	0.0

The mean (sd) healthcare costs for cases and controls over the five-year period were £1,196 and £337 for year 2, £1,148 and £316 for year 3, £1,126 and £325 for year 4, and £1,112 and £361 for year 5, respectively (Table 4).

**Table 4:** Total NHS healthcare costs for cases and controls for the first five years following index date

**Age group**	**Year**	**Group**	**Mean**	**Standard deviation**	**Median**	**Percentile 25**	**Percentile 75**
All ages	Y1	Case	£1,327	£2,114	£890	£427	£1,742
		Control	£328	£2,248	£69	£0	£214
	Y2	Case	£1,196	£2,228	£770	£302	£1,544
		Control	£337	£2,215	£65	£0	£208
	Y3	Case	£1,148	£3,749	£735	£267	£1,459
		Control	£316	£1,459	£64	£0	£197
	Y4	Case	£1,126	£3,535	£673	£235	£1,439
		Control	£325	£1,531	£64	£0	£201
	Y5	Case	£1,112	£4,137	£632	£196	£1,420
		Control	£361	£2,103	£65	£0	£211
Aged 6 to 17 years at index date	Y1	Case	£1,290	£2,119	£879	£425	£1,689
		Control	£315	£2,354	£64	£0	£198
	Y2	Case	£1,162	£2,195	£753	£296	£1,506
		Control	£333	£2,332	£64	£0	£199
	Y3	Case	£1,124	£3,917	£708	£264	£1,428
		Control	£308	£1,506	£62	£0	£191
	Y4	Case	£1,116	£3,712	£664	£236	£1,412
		Control	£325	£1,575	£64	£0	£201
	Y5	Case	£1,105	£4,377	£612	£186	£1,385
		Control	£372	£2,227	£65	£0	£213
Aged ≥18 years at index date	Y1	Case	£1,844	£2,118	£1,185	£648	£2,365
		Control	£550	£1,460	£130	£31	£427
	Y2	Case	£1,450	£1,616	£1,111	£385	£1,969
		Control	£509	£1,091	£116	£23	£419
	Y3	Case	£1,455	£2,157	£886	£546	£1,597
		Control	£604	£1,388	£111	£0	£422
	Y4	Case	£1,512	£2,077	£894	£417	£1,873
		Control	£660	£2,087	£96	£0	£372
	Y5	Case	£1,401	£1,439	£1,058	£265	£2,136
		Control	£515	£952	£118	£19	£495

## Discussion

In this retrospective study, the prevalence of diagnosed ADHD was notably lower than previously reported. We estimated that in 2009 the incidence of ADHD was 9.9 per100k population and the prevalence 81.5 cases per100k. Compared to a matched control group, those with ADHD had substantially increased resource use and related financial costs (four-fold).

A systematic review and meta-analysis characterising the worldwide prevalence of ADHD reported that the pooled prevalence was 5.3%, with significant variability [35]. In the UK in 1999 in children aged 5–15 years, the actual prevalence of ADHD—when estimated using the Development and Well-Being Assessment (DAWBA)—was 3.6% in boys and 0.9% in girls [1]. The difference between these two figures may be related to the sensitivity of the DAWBA compared with other diagnostic instruments. At 0.44% in boys and 0.05% in girls the estimates of prevalence of diagnosed ADHD in 1999 in children (6–17 years) in our study was much lower than either of these. The most likely explanation for this is that the epidemiological studies screened the population and aimed to identify both diagnosed and undiagnosed cases. In the UK only a minority of patients with ADHD currently seek or receive medical treatment for their condition [36,37]. The reason for the under-diagnosis of ADHD in the UK [38] is likely to be multifactorial. For example, parents of children with ADHD are likely to identify a problem and consult education professionals, but the presentation to primary care is limited and less than one in three children with ADHD access specialist services [37]. In addition, there is limited recognition of children at risk of ADHD in primary care [36] and uncertainty among many GPs over whether ADHD should be classed as medical disorder [39]. Even in the USA, where ADHD has been recognised longer, it was estimated that, between 2001 and 2004, less than half of the children meeting DSM-IV criteria received treatment [40]. In contrast to this, the percentage of children in the USA aged 4–17 years with a parent-reported ADHD diagnosis increased from 7.8% to 9.5% between 2003 and 2007 [41]. As the prevalence and incidence figures for this study relate to diagnosed ADHD, it is possible that any change in incidence or prevalence rates during the study period is an ascertainment effect.

The figures reported here are similar to those reported in a government-sponsored audit of ADHD services in Scotland [42]. In 2012, the overall prevalence had increased slightly to 0.7% with a similar variation across regions of Scotland and no change in the male-to-female ratio [42]. A UK study using the General Practice Research Database (GPRD; forerunner of CPRD) estimated that the prevalence of treated ADHD for patients aged 15–21 years was 0.88 per 1,000 in 1999, increasing to 5.09 per 1,000 in 2006 [43]. A slightly higher prevalence, though in a different age range, was reported by another study: 2.6 and 5.5 per 1,000 for 1999 and 2006, respectively, in patients aged 6–17 years [43].

We found that diagnosed cases of ADHD were more common in males than in females. Epidemiological studies have also reported a greater prevalence in males, with a male-to- female ratio of 2–3:1 [35]. In adults, however, the male-to-female ratio for ADHD has been reported to be approximately equal [44]. The higher ratios reported here and in other studies of diagnostic prevalence or treatment suggest that, in the UK, girls with ADHD are even less likely to be recognised and diagnosed than boys. It is possible that this is at least partly due to the fact that that females present with different symptoms and, most importantly, that they are less likely to have coexisting oppositional or disruptive behaviours [45]. However, a firm consensus on this matter has not been reached [16].

In our study, the diagnosed prevalence of ADHD in children age 6 – 17 years old increased from 192.4 to 506.4 per100k between 1998 and 2007. An increasing incidence rate was also observed between 1998 (39.3 per100k) and 2007 (79.0 per100k). An increase in the prevalence of ADHD has been reported in the USA between 1997 and 2007 [41,46]. Since 2007, the incidence and prevalence rates have decreased, suggesting that recognition rates may have peaked for the time being. This is broadly in line with the findings of the most recent NHS Scotland audit [42] and coincides with the publication of the NICE guidelines, although we do not expect this to have resulted in a decrease in the recognition of ADHD [16].

A systematic review with meta-analysis has suggested that the prevalence of ADHD declines with age (although the strict application of DSM-IV criteria designed for use in children may have led to an underestimation of prevalence in the adults) [47]. However, many people do continue to have significant ADHD-related impairments as adults [16]. A meta-analysis reported that the rate of persistence of a full DSM-IV diagnosis of ADHD was 15% at the age of 25 years, but when those patients fulfilling the DSM-IV definition of ADHD in partial remission were included, the rate of persistence increased to approximately 65% [48]. It has been estimated that this level of persistence equates to an estimated prevalence of 0.6–1.2% of adults by the age of 25 [16]. Our estimate of less than 0.02% prevalence in adults in 2009 (approximately 7,800 adults with ADHD in the UK [49]) is therefore much lower than expected [47,50], suggesting that the under-recognition of ADHD in adults exceeds that for children and adolescents. One possible explanation for this low prevalence rate could be that clinicians in the UK have only been diagnosing children over the last 20 years or so. As a consequence most adults were not diagnosed as children and, as services for adults are still not generally available, they are not yet getting diagnosed in large numbers as adults. Also, many adolescents are not transitioned to adult services. A study using data from GPRD identified that, for people aged 15–21 years between 1999–2006, prevalence of prescribing of ADHD medication decreased with increasing age but increased with increasing calendar year [43]. During the study period, we found a large increase in the prevalence of ADHD in adults (1.4 to 16.1 per 100,000 between 1998 and 2009), suggesting that either ADHD is now being increasingly recognised in adults or that children with a diagnosis of ADHD have grown and are still recognised to have the condition as adults.

The magnitude of the difference in annual mean costs was surprising. Prescription costs in year 1 were higher for cases compared to controls (£308 and £37, respectively), largely due to the cost of ADHD medicines. NICE guidance indicates that drug treatment should be first line when ADHD is severe and can be considered for moderate ADHD and impairment in school-aged children and young adults when non-pharmacological approaches are unsuccessful [16]. In adults, drug treatment is recommended by NICE as first line unless the patient prefers psychological treatment. Drug treatment is not recommended for pre-school children. Within the context of significant under-recognition it is likely that those individuals receiving a diagnosis would be at the more severe end of the ADHD continuum. As a consequence medication treatment would often be considered the first-line treatment for all except the very young.

Numerous studies investigating the healthcare costs associated with ADHD have been carried out in the USA, but their applicability to the UK NHS is questionable due to different patterns of service provision. Using information available for the UK, some estimates have been made of the cost of certain aspects of healthcare for ADHD at the population level. For health, social care, and educational services, it has been estimated that the NHS spends approximately £23 million on initial specialist assessment of ADHD in England and Wales and £14 million on follow-up care over one year [51]. In addition, the NHS spent approximately £8.5 million, £1.3 million, and £25.7 million on prescriptions for atomoxetine, dexamfetamine, and methylphenidate, respectively, in 2010 [3]. It is likely that almost all of this would have been spent in the treatment of ADHD, although dexamfetamine and methylphenidate also have an unlicensed indication for narcolepsy [21]. Furthermore, the mean annual cost of health and social care and educational resources relating to ADHD per adolescent in the UK has been estimated as £5,493 (median £2,327), where 24% of this cost relates to health [52]. In addition, ADHD commonly occurs with other conditions such as learning disorders, conduct and oppositional disorders, Tourette’s syndrome, bipolar disorder, anxiety and depression [16], and these conditions are likely to contribute to the higher healthcare costs observed for ADHD patients.

This study had inherent limitations. For cases where there was no prescription for an ADHD medication the requirement of two or more diagnoses was used in order to avoid selecting patients where the GP had recorded a provisional diagnosis of ADHD prior to referral for assessment by a specialist. However, this may have led to the exclusion of possible ADHD patients from the cost calculation and an underestimate of the incidence and prevalence rates. The care pathway for ADHD differs in comparison to many chronic conditions and will vary by site. Once the condition has been stabilised, GPs often prescribe drugs for ADHD under shared-care protocols. Prescriptions written in secondary care are not recorded in CPRD and could not be costed. Any underestimation of resource use and the related financial cost will disproportionately impact on the ADHD group and therefore the differences reported may underestimate the true difference. Those patients who are more difficult to stabilise may be less well recorded in CPRD as more of their healthcare may be provided in secondary care. In addition, our study index date may vary between patients from the date of first presentation to the GP, the date of referral back to the GP from secondary care, or the date of the first GP prescription for an ADHD medication. CPRD includes GP practices from all four UK regions and is therefore generalisable to the whole of the UK. On the other hand, the linked HES data is exclusively English, which could suggest that the healthcare cost estimates are generalisable to England only. However, the patients registered in the linked practices have been shown to be representative of the whole CPRD population [53].

Regarding the estimation of prevalence, a patient had to have received a diagnosis of ADHD or a prescription for a medication for ADHD both prior to and after 1st July each year. Although a washout period of 12 months was applied, the time between the mid-year point and the last collection date for the database becomes shorter for the more recent years and this may have contributed to the reduction in prevalence rates since 2007. However, this method was selected since clinical records in CPRD cannot be used to determine when a patient stops experiencing ADHD. The calculation of incidence is sensitive to the method used to calculate the denominator. Although, for this study, patients need not necessarily have had contact with their general practice to be included in the patient-years estimate. An underestimation in the incidence and prevalence rates could also have occurred if diagnoses were not accurately recorded in CPRD. The validity of medical diagnoses in CPRD have been confirmed in several studies [54,55]. GPs in the UK act as gatekeepers, and referrals to and outpatient letters from secondary care should be recorded in CPRD. However, ADHD diagnoses may be less well recorded than other conditions diagnosed and treated exclusively in primary care.

## Conclusion

In summary, the prevalence of diagnosed ADHD in the UK was notably lower than in reports that used screening. Costs in those with ADHD were more than four times higher than in those without ADHD.

## Abbreviations

ADHD: Attention deficit/hyperactivity disorder; CPRD: Clinical Practice Research Datalink; DSM: Diagnostic and Statistical Manual of Mental Disorders; GP: General Practitioner; HRG: Healthcare Resource Group; NHS: National Health Service; NIC: Net ingredient cost; NICE: National Institute for Health and Care Excellence; PCA: Prescription Cost Analyses; PSSRU: Personal Social Services Research Unit.

## Competing interests

CLlM and CDP have been and SEH and SJJ are employed by Pharmatelligence, a research consultancy receiving funding from pharmaceutical companies. SEH is employed by Alliance Boots. CDP has consulted for the following manufacturers of diabetic pharmaceuticals: Astellas, BMS, Ferring, Lilly, Medtronic, Novo Nordisk, Sanofi-Aventis, and Wyeth. DC has received research grants from various health-related organisations, including European Union FP7, the National Institute for Health Research, Shire, and Vifor; consults for Shire; has been on advisory boards for Flynn Pharma, Janssen, Lilly, Medice, Novartis, Shire, and Vifor; has received royalties from Oxford University Press; and has received payment for lectures from Flynn Pharma, Janssen, Lilly, Shire, and Vifor. CJC has received research grants from various health-related organisations including Abbott, Astellas, Diabetes UK, the Engineering and Physical Sciences Research Council, the EASD, Ferring, GSK, Lilly, the Medical Research Council, Medtronic, MSD, the National Health Service, Pfizer, Sanofi-Aventis, Shire, and Wyeth; and consults for Amylin, Aryx, Astellas, Boehringer Ingelheim, BMS, Diabetes UK, Eisel, Ferring, GSK, Ipsen, Lilly, Medtronic, MSD, Pfizer, Sanofi-Aventis, Takeda, and Wyeth.

## Authors’ contribution

The authors contributed the following: CJC conceived the study. CJC, CDP, CLlM, and SEH contributed to study design. SEH and CLlM analysed the data. SEH, CJC, DC, and CDP interpreted the data. SEH drafted the manuscript. SJJ provided data preparation and technical support. CJC, SEH, and DC were involved in the writing and reviewing of the report. CJC had overall responsibility for the study and is overall guarantor. JS and PH of Shire Development LLC provided comments on the outline and the initial draft of the manuscript, but the final content of this manuscript, the ultimate interpretation, and the decision to submit it for publication to the Child and Adolescent Psychiatry and Mental Health was made by the authors independently. All authors, external and internal, had full access to all of the data (including statistical reports and tables) in the study and can take responsibility for the integrity of the data and the accuracy of the data analysis. All authors read and approved the final manuscript.

## Funding

This research was funded by Shire Development LLC.

## Supplementary Material

Additional file 1: Figure S1 Study Numbers. Click here for file
