# Supplementary material for: The prevalence and incidence, resource use and financial costs of treating people with attention deficit/hyperactivity disorder (ADHD) in the United Kingdom (1998 to 2010)
Source: Child Adolesc Psychiatry Ment Health. 2013 Oct 11;7:34. doi: 10.1186/1753-2000-7-34 (PMC3856565; doi:10.1186/1753-2000-7-34)
Supplement: Additional file 1: Figure S1 — Study Numbers. [file 1753-2000-7-34-S1.doc]

**Additional file 1: Figure S1. Study Numbers**

Where accept status in CPRD is 1

Patients with a diagnosis for ADHD but not on treatment for ADHD = 11,137

Patients with diagnosis of ADHD and on treatment for ADHD = 11,979

Patients on treatment for ADHD but no ADHD diagnosis = 4,852

Patients with >=2 diagnoses for ADHD, no Rx for ADHD medication and CPRD accept status is 1 = 1,464

Patients with >=1 diagnosis ADHD and >=1 Rx ADHD medication = 11,979

Total = 13,443

Patients with no diagnosis of narcolepsy = 13,436

7 patients excluded as they had a diagnosis for narcolepsy

9,673 excluded as only received one diagnosis for ADHD and no Rx for ADHD medication and 4,852 excluded as only received Rx ADHD medication and no ADHD diagnosis

Patients with suitable dates = 13,325

Patients excluded because date of onset of ADHD unknown = 92

Patients excluded because date of first ADHD drug Rx > data censor date (earliest of transfer-out, death and data-collection dates) = 14

Patients excluded because date of first ADHD diagnosis > censor date = 5

Patients with adequate wash-in period = 8,063

Patients deleted because wash-in from registration date to index date < 180 days = 5,262

Patients adequately matched with 3 control patients = 8,058

Patients excluded as matched with <3 control patients = 5

Patients excluded as less than one year follow-up in CPRD = 589

Patients with at least one year follow-up in CPRD = 7,469

Patients excluded as less than one year follow-up in CPRD-linked HES = 3,755

Patients with at least one year follow-up in CPRD- linked HES = 3,233

Patients excluded as multiple prescriptions issued on the same date for same medication = 4

Patients included in the study= 3,229
